# Total-Body Parametric Imaging Using Relative Patlak Plot

**DOI:** 10.2967/jnumed.124.268496

**Published:** 2025-04

**Authors:** Siqi Li, Yasser G. Abdelhafez, Lorenzo Nardo, Simon R. Cherry, Ramsey D. Badawi, Guobao Wang

**Affiliations:** 1 Department of Radiology, University of California Davis Medical Center, Sacramento, California; and; 2 Department of Biomedical Engineering, University of California at Davis, Davis, California

**Keywords:** total-body PET parametric imaging, kinetic modeling, relative Patlak plot, standard clinical scan, self-supervised deep learning

## Abstract

The standard Patlak plot, a simple yet efficient model, is widely used to describe irreversible tracer kinetics for dynamic PET imaging. Its widespread application to whole-body parametric imaging remains constrained because of the need for a full-time-course input function (e.g., 1 h). In this paper, we demonstrate the relative Patlak (RP) plot, which eliminates the need for the early-time input function, for total-body parametric imaging and its application to 20-min clinical scans acquired in list mode. **Methods:** We conducted a theoretic analysis to indicate that the RP intercept *b*′ is equivalent to a ratio of the SUV relative to the plasma concentration, whereas the RP slope *K_i_*′ is equal to the standard Patlak *K_i_* (net influx rate) multiplied by a global scaling factor for each subject. One challenge in applying RP to a short scan duration (e.g., 20 min) is the resulting high noise in the parametric images. We applied a self-supervised deep-kernel method for noise reduction. Using the standard Patlak plot as the reference, the RP method was evaluated for lesion quantification, lesion-to-background contrast, and myocardial visualization in total-body parametric imaging in 22 human subjects (12 healthy subjects and 10 cancer patients) who underwent a 1-h dynamic ^18^F-FDG scan. The RP method was also applied to the dynamic data reconstructed from a clinical standard 20-min list-mode scan either at 1 or 2 h after injection for 2 cancer patients. **Results:** We demonstrated that it is feasible to obtain high-quality parametric images from 20-min scans using RP parametric imaging with a self-supervised deep-kernel noise-reduction strategy. The RP slope *K_i_*′ was highly correlated with the standard Patlak *K_i_* in lesions and major organs, demonstrating its quantitative potential across subjects. Compared with conventional SUVs, the *K_i_*′ images significantly improved lesion contrast and enabled visualization of the myocardium for potential cardiac assessment. The application of the RP parametric imaging to the 2 clinical scans also showed similar benefits. **Conclusion:** Using total-body PET with the RP approach, it is feasible to generate parametric images using data from a 20-min clinical list-mode scan.

## Visual Abstract

**Figure:**
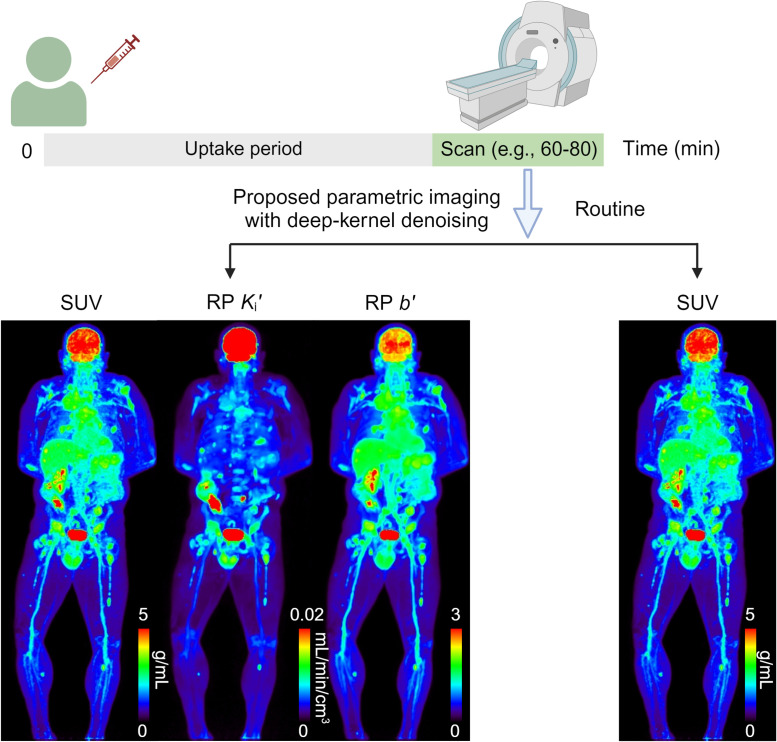

Dynamic ^18^F-FDG PET with tracer kinetic modeling enables multiparametric imaging and provides more accurate metabolic information as compared with static imaging (1,2). Among various kinetic modeling approaches, the Patlak graphical plot (3) is a commonly used linear kinetic model to describe ^18^F-FDG kinetics. The slope parameter of the standard Patlak plot, *K_i_*, represents the ^18^F-FDG net influx rate and has demonstrated advantages beyond the SUV images, for example, for improving tumor detection and discrimination (4–6), monitoring response to treatment (7,8), and evaluating the prognostic outcome (9,10).

Whole-body parametric imaging with the standard Patlak plot has been implemented on conventional PET scanners with a short axial field of view (AFOV) ranging from 15 to 30 cm using a multibed and multipass acquisition strategy (6,11). The advent of long-AFOV and total-body PET scanners with a much longer AFOV (>1 m), such as the UIH uEXPLORER (United Imaging) (12,13) and the Siemens Quadra (Siemens Healthineers) (14), has further simplified and improved the implementation of Patlak parametric imaging because of the much-improved detection sensitivity and simultaneous coverage of multiple organs. However, all these methods require a full-time-course dynamic scan (e.g., 1 h) to obtain an image-derived input function, which limits their broad use. Although a population-based input function (PIF) may be used (15,16), it is challenging to adapt the approach to individual patients, particularly for those with diabetes or impaired renal function (17) and for pediatric patients (18).

The relative Patlak (RP) plot (19), which does not require the early-time input function but only the late-time input function, is another solution to streamline the parametric imaging process. This method has been recently deployed on commercial short-AFOV scanners for whole-body parametric imaging (11). The RP slope *K_i_*′ is equivalent to the standard Patlak *K_i_* multiplied by a global scaling factor, thus providing comparable performance for lesion detection (19). However, its potential for absolute quantitation has not been demonstrated. Early implementation of the RP plot also commonly used a scan duration of 30 min or more (11), requiring data acquisition longer than a typical clinical scan duration (commonly up to 20 min) (20).

This paper aims to develop and evaluate the potential of the RP plot for total-body parametric imaging from the dynamic data of a clinical 20-min scan. This method will add a new ability on top of standard clinical imaging to generate parametric images from the same acquired list-mode data. One challenge here is that the noise level in the resulting parametric images will be higher because of the use of a shorter scan duration. We thus propose a self-supervised deep-kernel method to improve the quality of RP parametric imaging. The proposed total-body RP approach has the unique advantage of being usable for delayed scans (e.g., a 20-min scan at 1–2 h after injection) and for pediatric patients, for whom it is difficult to obtain a PIF.

## MATERIALS AND METHODS

### Standard and RP Plots

The standard Patlak plot (3) describes the linear relationship between the normalized tissue concentration and normalized integral of input function after an equilibrium time t*:

$$\frac{C_{T}\left(t\right)}{C_{p}\left(t\right)}=K_{i}\cdot \frac{\int_{0}^{t}C_{p}\left(\tau \right)d\tau }{C_{p}\left(t\right)}+b\;\left(t>t^{*}\right),$$ Eq. 1

where *C*_T_(*t*) denotes the ^18^F-FDG concentration in a tissue region at the time *t*. *C*_p_(*t*), also called input function, represents the ^18^F-FDG concentration in the plasma at the time *t*. *K_i_* is a slope parameter representing the ^18^F-FDG net influx rate. *b* is an intercept parameter representing a mixture of blood volume and free-state ^18^F-FDG volume of distribution in tissue (3). The 2 parameters can be estimated via linear regression. The common choice of t* for total-body Patlak parametric imaging is greater than or equal to 30 min (21). Note that although only the time–activity data of later than t* is required for *C*_T_(*t*), the plot still requires a full-time-course input function from the injection time to the end of the scan for *C*_p_(*t*).

The RP plot (19) was proposed as formulated in the following equation:

$$\frac{C_{T}\left(t\right)}{C_{p}\left(t\right)}={K\prime }_{i}\cdot \frac{\int_{t^{*}}^{t}C_{p}\left(\tau \right)d\tau }{C_{p}\left(t\right)}+b\prime \left(t>t^{*}\right),$$ Eq. 2

where *K_i_*′ and *b*′ are the new RP slope and intercept. Unlike the standard Patlak plot, the RP plot eliminates the need for the early-time input function from 0 to t* and only requires the late-time input function from t* to the end of the scan, thus potentially providing a more efficient approach for parametric imaging.

### Theoretic Interpretation of RP Plot

Our earlier study (19) demonstrated that the RP slope *K_i_*′ is equivalent to the standard Patlak *K_i_* multiplied by a global scaling factor α in each subject:

$${K\prime }_{i}=\alpha K_{i},$$ Eq. 3

leading to an equivalent spatial distribution between the parametric images of the two slopes.

The interpretation of the RP intercept *b*′, however, remains underexplored in previous studies. We note that Equation 2 leads to the following equation by setting *t* equal to t*:

$$\frac{C_{T}\left(t^{*}\right)}{C_{p}\left(t^{*}\right)}={K\prime }_{i}\cdot \frac{\int_{t^{*}}^{t^{*}}C_{p}\left(\tau \right)d\tau =0\;}{C_{p}\left(t\right)}+b\prime =b\prime ,$$ Eq. 4

which indicates that the intercept *b*′ is equivalent to the SUV ratio (SUVr) relative to the plasma input function at time t*:

$$b\prime =\mathrm{SUVr}\left(t^{*}\right)\equiv \frac{C_{T}\left(t^{*}\right)}{C_{p}\left(t^{*}\right)}.$$ Eq. 5

This equivalence suggests the RP intercept *b*′ is not arbitrary but has a physiologic interpretation, further expanding theoretic aspects of the RP plot.

### RP Parametric Imaging with Self-Supervised Deep-Kernel Denoising

One potential application of the RP plot is to shorten the scan duration for parametric imaging by increasing t*. Our preliminary analysis shown in Supplemental Figure 1 suggests that the noise level of *K_i_*′ image will be higher if the scan duration is shortened (supplemental materials are available at http://jnm.snmjournals.org). We thus applied the deep-kernel method (22) to overcome the noise issue.

A denoised image $x_{\mathrm{denoised}}\;$can be modeled using a generalized kernel representation:

$$x_{\mathrm{denoised}}=K\left(\theta ;Z\right)x_{\mathrm{noise}},$$ Eq. 6

which may also be explained as a type of nonlocal mean denoising. $x_{\mathrm{noise}}$ is a noisy image. $K\left(\theta ;Z\right)$ is a kernel matrix built on the image prior data ***Z*** with ***θ*** including any parameters that determine the kernel representation. Unlike the conventional kernel method (23) that uses an empirically defined $\theta_{e}$, the deep-kernel method (22) enables an optimized and learned kernel matrix by extending the kernel representation (Eq. 6) into trainable neural networks. The kernel matrix $K_{l}\left(\theta_{l};Z\right)$ now includes the trainable parameter $\theta_{l}$ and will be trained using a simplified self-supervised strategy for deep-kernel learning through a denoising autoencoder framework. The model parameter set ***θ*** is estimated using the following least-square formulation to minimize the differences between noisy frames and their labels:

$${\hat{\theta }}_{l}=\mathrm{argmi}n_{\theta_{l}}\sum_{m=1}^{n_{z}}{\left|\left|z_{a}\text{−}K_{l}\left(\theta_{l};Z\right)z_{m}\right|\right|}^{2},$$ Eq. 7

where ${Z=\{z_{m}\}}_{m=1}^{n_{z}}$ consists of $n_{z}$ dynamic frames. $z_{a}$ is the corresponding mean image of all the frames, considered as the clean training label. In this work, $n_{z}=4$ and each $z_{m}$ was thus a 5-min frame. All the training settings (neighborhood number, 200; training iteration, 300; learning rate, 1 × 10^−3^) were the same as used previously (22). Once ${\hat{\theta }}_{l}$ is trained, $K_{l}({\hat{\theta }}_{l};Z)$ is then used on both dynamic activity images and parametric images for noise reduction.

### Total-Body Dynamic PET Data Acquisition for Validation

Twenty-two subjects, including 12 healthy volunteers and 10 patients with immunotherapy-naïve, metastatic genitourinary cancer, were included in this study and scanned using the uEXPLORER total-body PET scanner. Prior Ethics Committee and Institution Review Board approval and written informed consent were obtained. After a total-body CT scan, each participant underwent a 1-h dynamic scan with an injection of approximately 370 MBq of ^18^F-FDG. The resulting list-mode data were reconstructed into 29 frames of dynamic images (6 × 10 s, 2 × 30 s, 6 × 60 s, 5 × 120 s, 4 × 180 s, and 6 × 300 s) using vendor-implemented time-of-flight ordered-subset expectation maximization (OSEM) algorithm with 4 iterations and 20 subsets (13). The random correction, scatter correction, attenuation correction, deadtime correction, and decay correction were all applied. The image size of each frame was 150 × 150 × 486, and the voxel size was 4 × 4 × 4 mm^3^.

The RP plot was implemented on the late 20-min data of each 1-h dynamic scan (i.e., t* = 40 min) with an image-derived input function from a region of interest (ROI) placed in the ascending aorta (24). The standard Patlak plot was also applied to the same 20-min dynamic data but with a full 1-h blood input function. The SUV image was calculated using the last 5-min data. In total, 26 lesions were identified in genitourinary cancer patients. An ROI was also placed in major organs, including the liver, lungs, spleen, muscle, gray matter, and bone marrow of the spine and pelvis, leading to 154 organ ROIs in all subjects. The ROI locations of bone marrow and muscle are provided in supplemental materials. All the ROI delineation was performed using AMIDE software (25).

### Demonstration of RP Parametric Imaging with Deep-Kernel Noise Reduction

The RP parametric imaging derived from the OSEM reconstruction was considered the nondenoising reference. Different postreconstruction denoising methods, including the conventional kernel method (23), the 4-dimensional deep image prior (4D-DIP) method (26), and the deep-kernel denoising method, were compared for evaluation of the RP parametric *K_i_*′ imaging. They are all learning-based methods based on single subjects and do not require population-based pretraining. The implementations of the conventional kernel and 4D-DIP methods are provided in the supplemental materials. Different approaches were compared using the trade-off of lesion contrast recovery (*K_i_*′ value) versus the liver noise level calculated as the SD divided by the mean.

To understand how denoising methods affect the lesion ROI quantification accuracy of RP *K_i_*′, we calculated the percentage difference in *K_i_*′ between each denoising method and the nondenoising OSEM reference (Δ*K_i_*′ = (ROI_denoised_ − ROI_ref_)/ROI_ref_) for all lesions.

With the deep-kernel noise-reduction approach, we then verified the total-body global-scaling relationship between the RP slope *K_i_*′ image and the standard Patlak slope *K_i_* image, as well as the equivalence between the RP intercept *b*′ and the SUVr at t* using the scatter plots of all image voxels between the 2 methods. Linear regression was used to evaluate the correlation.

### Comparison of Total-Body *K_i_*′ Images with *K_i_* and SUV for Lesion Contrast and Myocardial Visualization in Cancer Patients

To demonstrate the potential benefit of total-body RP parametric imaging for lesion detection, the parametric images of *K_i_*′ were compared with SUV images using the standard Patlak *K_i_* images (derived using the full 1-h input function) as the reference. We computed the lesion-to-liver contrast ratio (CR), defined as ROI_lesion_/ROI_liver_, for *K_i_*′, SUV, and *K_i_* for all 26 lesions from 10 cancer patients. The paired *t* test and Wilcoxon signed-rank test were used to evaluate their statistical differences. *P* values of less than 0.05 were considered statistically significant. The CR difference between *K_i_*′ and SUV, defined as (${\mathrm{CR}}_{K_{i}^{\prime }}\text{−}{\mathrm{CR}}_{\mathrm{SUV}})/{\mathrm{CR}}_{\mathrm{SUV}}\times 100\%$, was calculated for each lesion. A positive value means the better contrast provided by *K_i_*′. In addition, parametric imaging has the advantage of better visualizing the myocardium than the SUV does, as shown in our early work (27). Thus, the 3 images were also compared for their ability to visualize the myocardium in cancer patients. Note that the SUVr images provide the same spatial appearance and lesion contrast as SUV does and are therefore not included in the comparison.

### Evaluation of Quantitative Potential of *K_i_*′ Using *K_i_* as Reference

Because there is a global scaling factor between the parametric image of RP *K_i_*′ and that of the standard Patlak *K_i_* in each subject, it was unclear whether the RP *K_i_*′ can be quantitative across subjects. We conducted a linear correlation analysis between *K_i_*′ and *K_i_* using the data of 154 organ ROIs and 26 lesion ROIs from the 22 subjects. The 95% CI and prediction interval of the linear fitting were also included. The coefficient of variation, defined as the ratio of the SD to the mean, was used to measure how discretely this global scaling factor α is between different subjects. In addition, we estimated the global scaling factor from 12 healthy subjects and then applied it to the *K_i_*′ images of 10 cancer patients for calibration. The difference in all 26 lesion ROIs between the calibrated RP *K_i_* and standard Patlak *K_i_* (reference) was quantified using the Bland–Altman plot.

We also compared the RP method with the PIF method. Following reference (16), the PIF was determined from the 12 healthy subjects and then applied to the 10 cancer patients in our study to generate the PIF-based *K_i_* images. Again, the difference in lesions between PIF-based *K_i_* and standard Patlak *K_i_* was evaluated using the Bland–Altman plot.

### Application to Clinical List-Mode Scans

We further applied the RP parametric imaging approach to 2 clinical 20-min list-mode ^18^F-FDG PET scans to demonstrate the potential of the method. One was for a lymphoma patient scanned from 60 to 80 min after injection, and the other was for a lung cancer patient scanned from 120 to 140 min after injection. The list-mode raw data of each scan were reprocessed to generate 4 5-min frames of dynamic data. Implementation details and reconstruction settings were the same as those used in the aforementioned validation study. Because there is no early-time input function data available, the standard Patlak plot was not applicable. Thirteen lesions (8 from the lymphoma patient and 5 from the lung cancer patient) were identified and used to evaluate the lesion contrast for *K_i_*′ and SUV. The paired *t* test was used to indicate statistical significance. All human subjects’ basic characteristics are summarized in Supplemental Table 1.

## RESULTS

### Total-Body RP Parametric Imaging Using Deep-Kernel Noise Reduction

**FIGURE 1.**
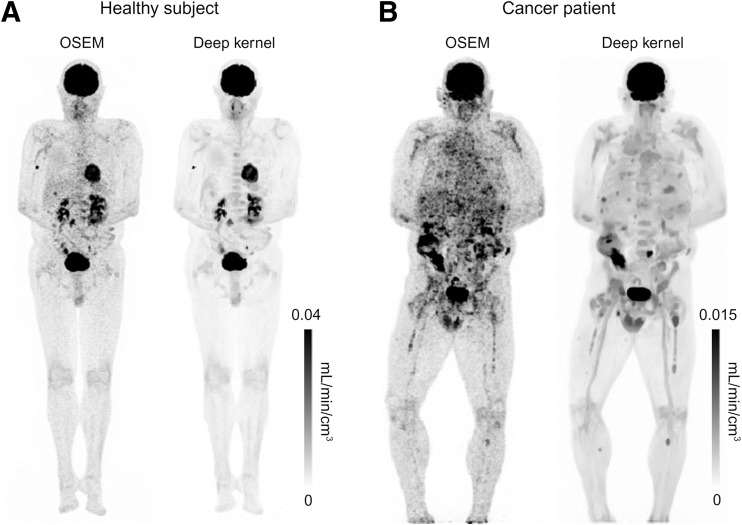
Total-body RP *K_i_*′ images from 20-min dynamic ^18^F-FDG scan (40–60 min after injection) based on standard OSEM reconstruction and deep-kernel noise-reduction method for healthy subject (A) and cancer patient (genitourinary cancer patient 3) (B). Images are shown in maximum-intensity projection.

Figure 1 shows the total-body parametric image of the RP slope *K_i_*′ (mL/min/cm^3^) generated from a 20-min dynamic scan for a healthy subject and a cancer patient using the standard OSEM reconstruction method (without denoising) and the deep-kernel noise-reduction method. Without postreconstruction noise reduction, the standard OSEM yielded a noisy *K_i_*′ image. The deep-kernel method substantially improved the *K_i_*′ image quality with lower noise and clearer lesion visualization.

Comparisons of the deep-kernel method with the conventional kernel, and 4D-DIP noise-reduction methods are presented in Supplemental Figures 2 and 3. Although the latter 2 methods both suppressed the noise as compared with the nondenoising OSEM reference (Supplemental Fig. 3A), they also significantly reduced lesion contrast and introduced relatively large ROI quantification differences (mean ± SD: conventional kernel, −16.9% ± 12.1%; 4D-DIP, −40.7% ± 17.72%). The deep-kernel method achieved a similar high lesion contrast as the OSEM reference did but also a much lower background noise as the 4D-DIP had (Supplemental Figs. 2C and 2D). The deep-kernel method also provided an equivalent ROI quantification of *K_i_*′, as indicated by the small differences (mean ± SD: −2.3% ± 2.7%) as compared with the nondenoising reference (Supplemental Fig. 3A), which holds for different lesion sizes (Supplemental Fig. 3B) in our study.

Of note, all subsequent parametric image visualizations and ROI analyses are based on the deep-kernel method.

### Demonstration of the Theoretic Aspects of Total-Body RP Parametric Imaging

**FIGURE 2.**
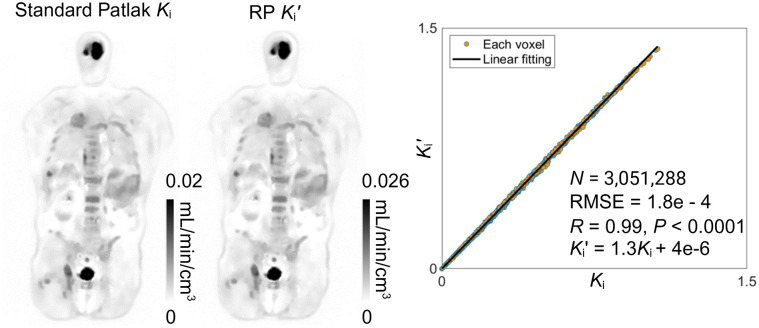
Demonstration of relationship between standard Patlak *K_i_* and RP *K_i_*′ for cancer patient (genitourinary cancer patient 3). From left to right: total-body *K_i_* image, total-body *K_i_*′ image; correlation plot of all image voxels. Global scaling factor was approximately 1.3. RMSE = root mean square error.

Figure 2 shows the comparison between the standard Patlak slope *K_i_* and RP slope *K_i_*′ images for a cancer patient. These 2 images were visually identical, though their absolute values are different. Their correlation plot verified that *K_i_*′ is equivalent to *K_i_* multiplied by a global scaling factor, that is, α = 1.3 for this subject. The *y*-intercept of the fitted line was nearly zero.

**FIGURE 3.**
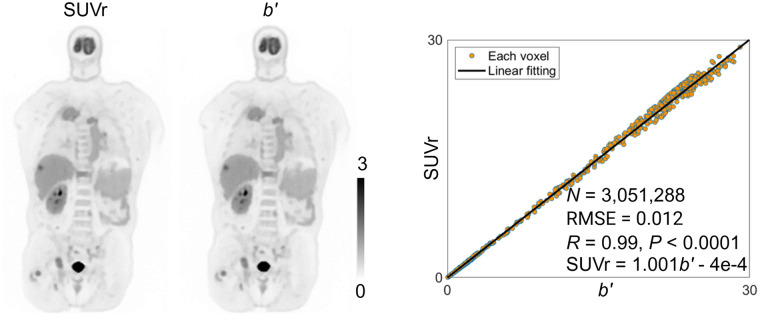
Demonstration of equivalence between SUVr (t* = 40 min) and RP intercept *b*′ in cancer patient (genitourinary cancer patient 3). From left to right: total-body SUVr image; total-body *b*′ image; correlation plot of all image voxels. Fitting line was almost identical to identity line. RMSE = root mean square error.

Figure 3 shows the comparison between the SUVr (t* = 40 min) and RP intercept *b*′ images for the same subject. The *b*′ image was closely equivalent to the SUVr image with an excellent fitting by the identity line. The linear correlation was statistically significant with a high correlation coefficient *R* (close to 1) and a minimal *P* value (<0.0001).

Tests on other subjects also showed similar results. These data together verified the theoretic relationships of the RP slope *K_i_*′ and intercept *b*′ with respect to the standard Patlak slope and SUVr, respectively.

### Comparison of RP *K_i_*′ with SUV for Lesion Contrast and Myocardial Visualization

**FIGURE 4.**
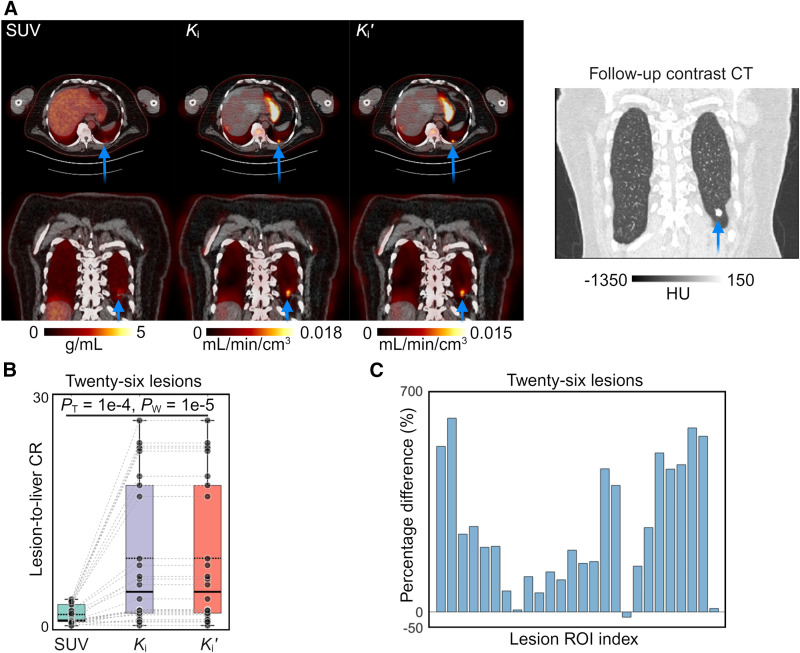
(A) Image comparison of lesion contrast between SUV, *K_i_*, and *K_i_*′ for cancer patient (genitourinary cancer patient 7). Fused images with CT are shown in transverse and coronal planes. Arrow points to lung metastasis as confirmed by follow-up contrast CT. (B) Boxplot comparison of lesion-to-liver CR for all 26 lesions for SUV, *K_i_*, and *K_i_*′. Paired lines, *P* values of paired *t* test (*P*_T_), and Wilcoxon signed-rank test (*P*_W_) are included. (C) Bar plot of percentage difference of CR between *K_i_*′ and SUV for each lesion. Note that CR difference between *K_i_*′ and *K_i_* is zero for each lesion and not shown. HU = Hounsfield unit.

Figure 4A shows the image comparison between SUV, *K_i_*, and *K_i_*′ for a cancer patient. A follow-up contrast CT image was included to confirm a lung metastasis indicated by the arrow. The lesions were clearly identified in both *K_i_* and *K_i_*′ images, whereas the signal was much weaker in the SUV image. The *K_i_*′ image showed a lesion-to-liver contrast of 2.5, which is the same as *K_i_* provided but much higher than the 0.59 provided by the SUV image.

Figure 4B further shows a group comparison of the lesion-to-liver CR of all 26 lesions in the 10 cancer patients. *K_i_*′ had a nearly 4-fold higher lesion contrast than SUV, with a median value of 4.85 versus 1.22. As indicated by the horizontal paired lines, *K_i_* and *K_i_*′ had exactly the same contrast results because the global scaling effect does not change the contrast.

The CR difference between *K_i_*′ and SUV for each individual lesion is further shown in Figure 4C. In 25 of the 26 lesions, the *K_i_*′ demonstrated a higher contrast, whereas the remaining one showed a slightly lower contrast than did the SUV.

**FIGURE 5.**
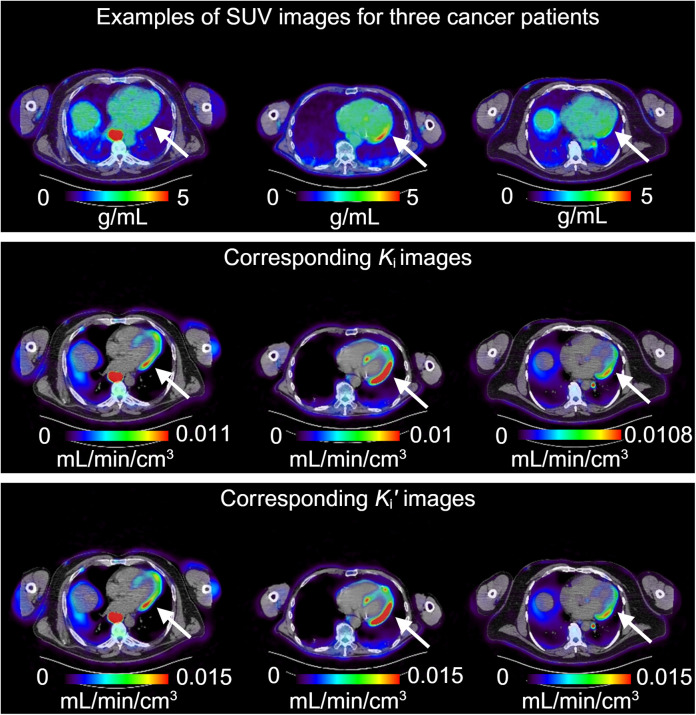
Comparison of parametric images with SUV images to visualize myocardium in 3 cancer patients (genitourinary cancer patients 1, 4, and 7). From top to bottom: SUV, standard Patlak *K_i_*, and RP *K_i_*′ images. All images were superimposed on CT. Arrows point to myocardium regions.

The top row of Figure 5 shows the SUV images of 3 cancer patients with a view of the heart region. The following 2 rows show their corresponding standard Patlak *K_i_* and RP *K_i_*′ images. Even though with different intensity ranges, both *K_i_* and *K_i_*′ images demonstrated a clear visualization of the myocardium (especially for the left ventricular myocardium), whereas the SUV images could not. This result suggests that RP parametric imaging can visualize and potentially characterize the myocardium in cancer patients.

### Quantitative Potential of RP *K_i_*′ Across Different Subjects

**FIGURE 6.**
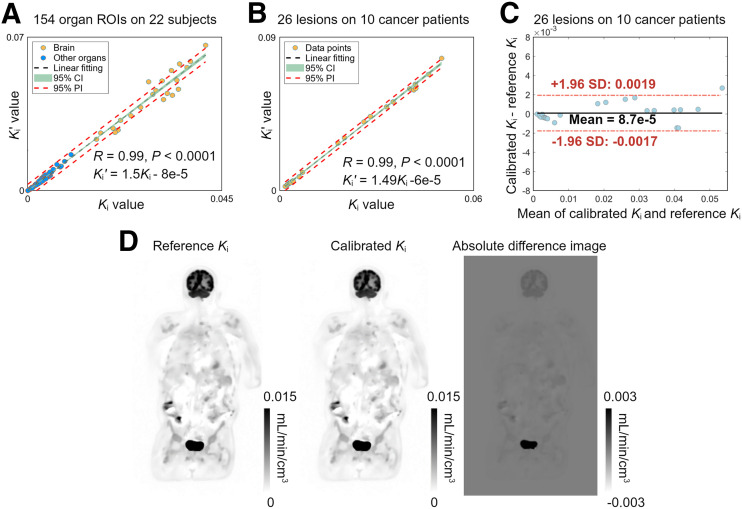
Demonstration of quantitative potential of RP *K_i_*′ compared with standard Patlak *K_i_*. (A) Correlation plot between standard *K_i_* and RP *K_i_*′ for 154 organ ROIs in 22 subjects. (B) Correlation plot between standard *K_i_* and RP *K_i_*′ for ROI quantification of 26 lesions. CI, prediction interval, *R*, and *P* values are also included. (C) Bland–Altman plot of lesion ROI quantification between reference *K_i_* from standard Patlak and calibrated *K_i_* from RP *K_i_*′. (D) Parametric images of reference *K_i_* and calibrated *K_i_* for cancer patient, as well as their absolute difference image (genitourinary cancer patient 3). PI = prediction interval.

Figure 6A shows the correlation plot for 154 organ ROIs from all 22 subjects. The intersubject correlation between *K_i_* and *K_i_*′ was strong, as indicated by a high correlation coefficient (*R* > 0.99), a minimal *P* value (<0.0001), and a narrow CI. Patient-wise the scaling factor was 1.47 ± 0.12 across these 22 subjects. Further analysis for each organ ROI (Supplemental Fig. 4) also indicated this strong correlation behavior (all *R* ≥ 0.95 and *P* < 0.0001). Organ-wise the scaling factor was 1.51 ± 0.03 across different organs.

Figure 6B shows the correlation between *K_i_* and *K_i_*′ for ROI quantification of 26 lesions in 10 cancer patients. Again, the intersubject correlation between *K_i_* and *K_i_*′ was statistically significant with a high correlation coefficient (*R* = 0.99) and a narrow CI.

Figure 6C shows the Bland–Altman plot of the calibrated *K_i_* from *K_i_*′ as compared with the standard Patlak *K_i_*. The mean difference (solid line) was close to 0 (8.7 × 10^−5^) and the difference of the 2 measures was within the limits of agreement (dashed line) for lesion ROIs (−0.0017 to 0.0019). Figure 6D further shows the parametric images of the standard Patlak *K_i_* and calibrated *K_i_* for a cancer patient, as well as the difference image in absolute, demonstrating a minimal difference.

The difference in all lesions between PIF *K_i_* and standard Patlak *K_i_* is presented in Supplemental Figure 5A. Both the RP method (after calibration) and PIF method indicated strong agreement with the standard Patlak method for lesion quantification. The RP approach exhibited a slightly better performance than the PIF method, as demonstrated by the narrower limit of agreement. A specific example of *K_i_* image is further provided in Supplemental Figure 5B, showing the PIF method overestimated the lesions, whereas the RP method stayed close to the reference.

### Application of RP Parametric Imaging to Clinical 20-Minute Scans

**FIGURE 7.**
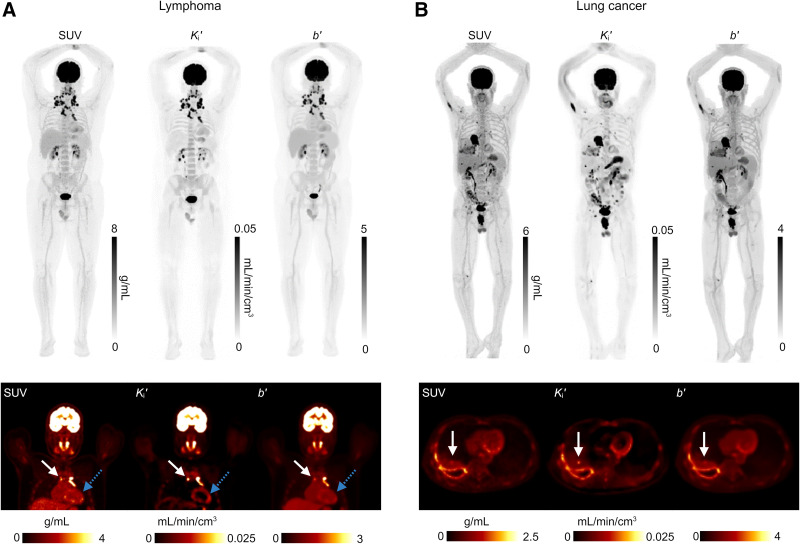
Total-body RP parametric imaging for 2 clinical scans and comparison to SUV images. (A) Lymphoma patient scanned from 60 to 80 min after injection. (B) Lung cancer patient scanned from 120 to 140 min after injection. Images in top row are shown with maximum-intensity projection. More detailed comparison for lesions or myocardium is shown in bottom row. Solid arrows (left, lymph node; right, pleural nodule) indicate improved lesion contrast by *K_i_*′, and dashed arrows demonstrate ability of *K_i_*′ for better myocardium visualization.

Figure 7 shows the total-body RP parametric images generated from 2 clinical 20-min ^18^F-FDG scans, 1 for a lymphoma patient scanned from 60 to 80 min and the other for a lung cancer patient scanned from 120 to 140 min. Their SUV images are also shown for comparison. Although the RP *b*′ image exhibited information comparable to that of the SUV image, the *K_i_*′ images showed improved lesion contrast and better visualization of the myocardium. Table 1 further shows a quantitative comparison of lesion-to-liver contrast for 13 lesions in the 2 patients. The lesion contrast improvement was statistically significant (*P* = 0.001).

**TABLE 1.** Comparison of Lesion Contrast for 13 Lesions in Two Patients Between SUV and *K_i_*′

Contrast	SUV	${K\prime }_{i}$	*P*_T_
Lesion	3.0 ± 1.9	11.0 ± 8.9	0.001

*P*_T_ = *P* value of paired *t* test.

## DISCUSSION

In this work, we demonstrated an efficient total-body parametric imaging approach using the RP plot that can be generated from a standard clinical acquisition and does not need the early-time input function. With the deep-kernel noise-reduction strategy, it becomes feasible to generate RP parametric images from a 20-min scan (Fig. 1; Supplemental Fig. 2). Compared with earlier work (19), this paper also demonstrated that the RP intercept *b*′ is equivalent to SUVr at the time t* (Eq. 5; Fig. 3), which offers a better understanding of the theoretic aspects of the RP plot.

The results of total-body parametric images from this work further verified that the RP slope *K_i_*′ image is equivalent to the standard Patlak *K_i_* image multiplied by a global scaling factor in each subject. This equivalence makes *K_i_*′ equal in utility to *K_i_* for those tasks that are generally not affected by a global factor, such as lesion detection and tumor volume segmentation. Compared with SUVs, a vital benefit of *K_i_*′ (and *K_i_*) was the improved lesion contrast (Fig. 4). Additionally, whereas the SUV images acquired at 1 h after injection were unable to show the myocardium clearly in 3 of 10 in our cancer cohort, the *K_i_*′ image enabled better visualization of the myocardium for potential cardiac assessment (Fig. 5), which has the potential to assess cardiotoxicity in cancer treatments (e.g., chemotherapy or immunotherapy). The influence of motion on accurate quantification of total-body parametric imaging, particularly in the heart and lung, is a significant consideration (28). We will investigate methods for mitigating motion for total-body RP parametric imaging in the future (29).

Furthermore, our pilot study of 22 subjects demonstrated a strong correlation between *K_i_* and *K_i_*′ across subjects for ROI quantification of lesions and major organs (Fig. 6). The coefficient of variation of the scaling factor was relatively small (8.3%) in this cohort. These results suggest the potential to use RP *K_i_*′ as a quantitative metric and warrant a future study with a large sample size to assess the test–retest performance and evaluate the potential of *K_i_*′ for tumor staging and therapeutic response assessment.

We further demonstrated the feasibility and benefits of applying the proposed total-body RP parametric imaging approach to standard clinical scans (Fig. 7). The improved lesion contrast and myocardial visualization may facilitate the potential integration of this efficient parametric imaging approach into standard clinical workflows. Of note, the RP *K_i_*′ is not aiming to replace SUVs but rather provides additional useful information without adding imaging time and scan costs.

Not limited to total-body PET, the RP parametric imaging method can also be extended to conventional shorter scanners using a multibed and multipass strategy (6,11). The noise in the parametric images may be a challenge but could be overcome by advanced reconstruction methods (30). Another application of the proposed approach would be for pediatric parametric imaging because a long (e.g., 1 h) dynamic scan is generally impractical for pediatric patients. A short scan with a PIF method may be used but is challenging because of the lack of a representative dataset for this population. In contrast, the RP can be a feasible solution and will be explored in the future.

There are limitations with this work. The scan duration of dynamic data was 20 min with 5 min for each frame. There is a growing trend toward shorter clinical scans, such as 10 min or less (31). A recent study demonstrated the feasibility of applying the standard Patlak plot to a 10-min scan through direct reconstruction and the PIF (32). Our future work will extend the RP method for scans of 10 min or shorter and also evaluate the best possible framing protocol. Concurrently, the consideration of motion correction is also warranted. The use of advanced noise-reduction algorithms may potentially introduce texture patterns in RP parametric images. It is thus worth further assessing the reliability of the generated images, such as for quantitative lesion detectability using a physician observer in future studies. The lesion CR was calculated using the liver as the background, not regional backgrounds that are more specific to the lesions, though the latter approach has its own limitations. In addition, this study indicates a relatively small variation in the scaling factor α across subjects, thus demonstrating the quantitative potential of RP *K_i_*′. However, this was limited to a single center and did not include a test–retest component. The variation may become larger in multicenter studies in which the injection protocols could be different. A future study would be needed to explore more in this direction.

Technically, both RP and PIF methods may introduce quantification bias compared with the standard Patlak plot because of the absence or mismatch of the early-phase input function. However, as shown by the reasonably good correlation between RP *K_i_*′ and standard Patlak *K_i_* across subjects (Fig. 6; *R* = 0.99), the RP *K_i_*′ may have potential to offer intersubject quantification even though they are not equal to the standard Patlak slope. The results demonstrated in this paper warrant a future study with a large sample size. We are investigating potential clinical value of the RP approach in addition to SUV on a larger lymphoma cohort which includes both adult and pediatric patients. We plan to evaluate the sensitivity and specificity of the RP approach for differentiation between benign and malignant lesions and the performance of quantification for tumor staging.

## CONCLUSION

In this paper, we have developed and implemented an efficient total-body parametric imaging approach using the RP plot and self-supervised deep-kernel noise reduction for dynamic ^18^F-FDG scans of 20-min duration acquired on the uEXPLORER total-body PET scanner. The RP *K_i_*′ was highly correlated with standard Patlak *K_i_* for ROI quantification across subjects, demonstrating a strong quantitative potential. The method can be used to enable parametric imaging from routine clinical scans and has the potential to be applied to late-time scans and to produce parametric images for pediatric patients who cannot tolerate a long dynamic scan duration.

## DISCLOSURE

This work was supported in part by NIH grants R01 EB033435 and R01 CA206187. The study was also supported by In Vivo Translational Imaging Shared Resources with funds from NCI P30CA093373. The University of California, Davis, has a research agreement and revenue-sharing agreement with United Imaging Health Care. No other potential conflict of interest relevant to this article was reported.

## Notes

KEY POINTS **QUESTION:** Current parametric imaging with dynamic ^18^F-FDG PET commonly uses a scan duration of 30–60 min. Is there a way to achieve total-body parametric imaging from clinical scans that acquire data for only 20 min starting 1–2 h after tracer injection? **PERTINENT FINDINGS:** The RP plot, in combination with a deep-kernel noise-reduction method, was shown to be capable of generating high-quality parametric images from a 20-min clinical scan and showed benefits over the standard SUV images. **IMPLICATIONS FOR PATIENT CARE:** The proposed method offers a new solution to achieve parametric imaging from a scan duration that is similar to current clinical static scans. It has the potential to apply to late-time scans and for parametric imaging of pediatric patients who cannot tolerate a long dynamic scan duration.
